# B-Cell Receptor Signaling and Beyond: The Role of Igα (CD79a)/Igβ (CD79b) in Normal and Malignant B Cells

**DOI:** 10.3390/ijms25010010

**Published:** 2023-12-19

**Authors:** Anton Tkachenko, Kristyna Kupcova, Ondrej Havranek

**Affiliations:** 1 BIOCEV, First Faculty of Medicine, Charles University, Prumyslova 595, 252 50 Vestec, Czech Republic; 2 First Department of Internal Medicine–Hematology, General University Hospital and First Faculty of Medicine, Charles University, 128 08 Prague, Czech Republic

**Keywords:** B lymphocytes, B-cell receptor, BCR, CD79a, CD79b, BCR signaling, BCR assembly, lymphoid malignancies, non-Hodgkin lymphoma, NHL, CLL, ITAM, immunoglobulin, malignant B cells, B cell development, antigen-induced BCR signaling, tonic BCR signaling, DLBCL, LYN, BTK, BCR internalization

## Abstract

B-cell receptor (BCR) is a B cell hallmark surface complex regulating multiple cellular processes in normal as well as malignant B cells. Igα (CD79a)/Igβ (CD79b) are essential components of BCR that are indispensable for its functionality, signal initiation, and signal transduction. CD79a/CD79b-mediated BCR signaling is required for the survival of normal as well as malignant B cells via a wide signaling network. Recent studies identified the great complexity of this signaling network and revealed the emerging role of CD79a/CD79b in signal integration. In this review, we have focused on functional features of CD79a/CD79b, summarized signaling consequences of CD79a/CD79b post-translational modifications, and highlighted specifics of CD79a/CD79b interactions within BCR and related signaling cascades. We have reviewed the complex role of CD79a/CD79b in multiple aspects of normal B cell biology and how is the normal BCR signaling affected by lymphoid neoplasms associated *CD79A*/*CD79B* mutations. We have also summarized important unresolved questions and highlighted issues that remain to be explored for better understanding of CD79a/CD79b-mediated signal transduction and the eventual identification of additional therapeutically targetable BCR signaling vulnerabilities.

## 1. Introduction

B-cell receptor (BCR) signaling plays a critical role at multiple stages of B cell life cycle [1]. BCR is a hallmark molecule of B cells that determines cellular fate and regulates B cell survival, fitness, activation, development, and transformation into immunoglobulin-secreting cells [2]. Structurally, BCR is composed of a membrane-bound immunoglobulin (Ig) molecule noncovalently linked with Igα (CD79a) and Igβ (CD79b) transmembrane signaling subunits [2,3,4]. BCR has a central role within the adaptive immune system and antigen recognition. Antigen binding triggers antigen induced BCR signaling, activating an array of signaling cascades and cellular processes. These include BCR-mediated antigen capture, recognition, uptake, and processing (also making B cells effective antigen-presenting cells) [5]. Importantly, BCR is also a source of baseline antigen-independent signaling, the so-called “tonic” BCR signaling. Tonic BCR signaling is necessary for correct B-cell development and for survival of normal mature B cells [6]. At the same time, altered BCR signaling has been implicated in the pathogenesis of autoimmune inflammatory disorders and B-cell derived malignancies [7,8,9,10].

In recent years, multiple studies have substantially improved our understanding of molecular mechanisms governing BCR signaling and highlighted the critical role of BCR signaling integrative components CD79a and CD79b. In this review, we focus specifically on functional features of the CD79a/CD79b heterodimeric unit in normal and malignant B cells, as well as on its role in BCR signal regulation. Furthermore, we summarize types and consequences of CD79a/CD79b heterodimer covalent posttranslational modifications, describe the CD79a/CD79b interaction network, and highlight CD79a- and CD79b-associated disruption of BCR signaling in malignant B cells. Reflecting the aim of our review, we focus specifically on signaling events related to CD79a and CD79b molecules, providing only a brief overview of BCR signaling in general.

## 2. BCR Signaling in Normal B Cells

BCR signaling is of critical importance for normal B cell performance at all different stages of their development. BCR can induce a wide array of cellular responses related to the complexity of the intracellular BCR signaling network. Individual features of BCR signaling are highly dependent on a specific stage of B cell development and activation status [11]. Importantly, pre-BCR, which is structurally similar to BCR but contains a surrogate light chain (SLC) made up of the invariant proteins λ5 (CD179b) and VpreB (CD179a) instead of the Ig light chain in BCR [12], is transiently expressed in early developmental stages and is necessary for pro-B to pre-B transition and pre-B cell expansion [13,14]. Correct assembly and proper SLC replacement with the conventional light chain in pre-BCR is an imperative for further B cell development [15].

BCR signaling is involved in the prevention of naïve B cell premature activation and expansion of autoreactive clones. At the same time, low-level “tonic” BCR signaling is essential for naïve B cell survival [16]. BCR antigen binding promotes mature B cell activation and further differentiation of naïve B cells via activation of PLC-γ2, PI3K/AKT and MAPK signaling pathways [17]. To ensure proper functionality of activated B cells, BCR signaling sustains survival, stimulates cell growth, and supports other related cellular adaptations via an array of signaling cascades including Ca^2+^ signaling, NF-κB activation, PI3K/AKT/mTOR, NFAT, ERK, and MAPK signaling [18]. Furthermore, BCR is vital for antigen presentation and subsequent T cell response activation and for B cell differentiation into antibody-producing plasma cells [19]. In particular, BCR-mediated antigen internalization is followed by intracellular antigen processing and subsequent surface presentation to CD4^+^ and CD8^+^ T cells [20]. BCR signaling also critically regulates activation-induced cytidine deaminase (AID)-mediated immunoglobulin class switch recombination [21,22]. Combination of activated BCR signaling with either a T cell-dependent (follicular T helpers) or T cell-independent (lipopolysaccharides or glycolipids) signal is crucial for B cell differentiation into antibody-secreting plasma cells or memory B cells [23]. BCR signaling driven plasma cell differentiation requires transcription factor Ets1 downregulation via Lyn-, PI3K-, BTK-, IKK2- and JNK-dependent pathways [24].

Additionally, BCR signaling contributes to the regulation of multiple other cellular processes in normal B cells including metabolism. For instance, it induces PI3K/AKT-dependent activation of glycolysis, oxidative phosphorylation, and glucose uptake [25,26]. BCR signaling also activates c-Myc with resulting enhancement of glycolysis and mitochondrial biogenesis [27]. BCR-initiated Ca^2+^ mobilization regulates metabolic reprogramming of naïve B cells which is required for their growth and further differentiation [18]. Maintenance of the balance between cellular growth and catabolic and anabolic processes is critical for correct B cell functionality and is primarily sustained via c-Myc and mTORC1 activity (which are both adjusted through BCR signaling) [28]. Importantly, BCR signaling was also implicated in metabolic regulation via autophagy upregulation [29]. BCR-mediated autophagy has been reported to be required for B cell activation [28].

Besides survival, activation, and proliferation, BCR signaling may prime B cells to anergy and cellular death to ensure B cell tolerance [30]. For instance, BCR signaling has been suggested to serve as a B cell quality control. Only moderate-intensity BCR signaling promotes positive selection, while BCR ligation downregulates BCR expression, reduces pro-survival PI3K/AKT signaling, and provides negative selection [31]. Inappropriately activated BCR may lead to B cell apoptosis [32]. BCR-mediated pro-apoptotic signaling has been associated with Ca^2+^-dependent and mitochondrial pathways [30].

Therefore, not only the type of BCR signaling, but also its intensity varies during B cell development and can determine the cell fate of B cells and their involvement in the immune response.

## 3. B-Cell Derived Malignancies and BCR Signaling

According to the recently updated 5th classification of lymphoid neoplasms (the World Health Organization Classification of Haematolymphoid Tumours), B-cell malignancies include the following categories: tumor-like lesions with B-cell predominance, precursor B-cell neoplasms (B lymphoblastic leukemias), mature B-cell neoplasms, and plasma cell neoplasms and other diseases with paraproteins [33]. Mature B-cell neoplasms include, e.g., pre-neoplastic and neoplastic small lymphocytic proliferations (e.g., chronic lymphocytic leukemia, CLL), multiple types of non-Hodgkin lymphomas (NHLs), and Hodgkin lymphomas [33]. The most common subtypes of NHL are diffuse large B-cell lymphoma (DLBCL) and follicular lymphoma (FL), diagnosed in approximately 25–30% and 20% of NHL patients, respectively [34,35,36,37]. In most cases, B-cell-derived tumors retain surface expression of BCR which variably supports malignant cell growth and survival [38,39]. BCR signaling has been shown to drive the growth and evolution of B-cell acute lymphoblastic leukemia (B-ALL), chronic lymphocytic leukemia (CLL), and multiple types of NHLs [12,40,41,42]. Pathogenic BCR signaling has been extensively studied and clearly demonstrated for DLBCL. Additionally, there is evidence that BCR supports tumor cell growth and survival in mantle cell lymphoma (MCL), FL, Burkitt’s lymphoma, and marginal zone lymphoma [43,44,45,46].

BCR signaling supports tumor cell growth and survival via various mechanisms. The first described tumorigenic mode of BCR signaling was the so-called “chronic active” BCR signaling triggered by self-antigen binding. Chronic active BCR signaling supports the viability and growth of malignant B cells mainly through the NF-κB signaling pathway [47,48]. Recently, it was shown that frequent lymphoma-associated mutations of MYD88 (myeloid differentiation primary response 88) adaptor protein lead to its spontaneous association with Toll-like receptor 9 (TLR9) and BCR, forming a My-T-BCR complex capable to trigger NF-κB activation [49]. Moreover, it was shown that lymphoma growth is also supported by antigen-independent, constitutive, lower intensity “tonic” BCR signaling. Tonic BCR signaling supports the growth and survival of tumor cells mostly via the PI3K/AKT/FOXO1 signaling pathway [50,51]. Antigen-independent cell autonomous BCR signaling with features of antigen-triggered BCR signaling was identified in CLL [40]. Importantly, in DLBCL, the type of BCR signaling (antigen driven or similar vs. tonic) reflects gene expression profiling-based cell-of-origin classification into the activated B cell like (ABC) DLBCL subtype and germinal center B cell like (GCB) DLBCL subtype, respectively [52].

Given the importance of BCR signaling in B-cell derived malignancies, its inhibition is one of the novel therapeutic approaches. It is represented mainly by three BTK (Bruton’s tyrosine kinase) inhibitors (ibrutinib, acalabrutinib, and zanubrutinib) approved and frequently used in the treatment of certain B-cell derived neoplasms. BTK inhibitors are effective; however, their toxicity and common resistance development represent substantial challenges that motivate the search for additional BCR signaling targeted inhibitors [53].

Important considerations regarding types of BCR signaling come from genomic studies, as documented in DLBCL. Distinct patterns of BCR signaling are reflected in tumor mutational patterns, which further expand the above-mentioned cell-of-origin DLBCL classification. Based on the spectrum of somatic alterations, genomic studies identified five to seven distinct genetic DLBCL subtypes [54,55,56,57,58]. The MCD (combined *MYD88^L265P^* and *CD79B* mutations), N1 (mutated *NOTCH1*), and A53 (aneuploid and *TP53* inactivation) subtypes are significantly overlapping with the ABC DLBCL subtype, whereas EZB (mutated *EZH2* and translocated *BCL2*), ST2 (mutated *SGK1* and *TET2*), and BN2 (translocated *BCL6* and mutated *NOTCH2*) are overlapping with GCB DLBCL [56]. Alternative classifications were published by Chapuy et al., including clusters 1 to 5 (BN2-DLBCL, A53-DLBCL, EZB-DLBCL, ST2-DLBCL, and MCD-DLBCL, respectively); and by Lacy et al., including MYD88, BCL2, SOCS1/SGK1, TET2/SGK1, and NOTCH2 clusters [55,58]. Pedrosa et al. later attempted to unite and simplify the existing classifications through the assessment of the mutational status of only 26 genes and BCL2 and BCL6 translocation status to facilitate their clinical implementation (two-step genetic DLBCL classifier; 2-S). The suggested 2-S subtypes are N1^2-S^, EZB^2-S^, MCD^2-S^, BN2^2-S^, and ST2^2-S^ [54]. Importantly, none of the above-mentioned genetic studies were able to assign all cases, leaving a substantial proportion of tumors unclassified. On the other hand, genetic studies provided insights into the contribution of CD79a and CD79b (and their mutations) towards tumorigenesis and lymphoma development [54,55,56,57,58].

## 4. Extracellular, Transmembrane, and Intracellular Domains of CD79a/CD79b Are Functionally Distinct in BCR

BCR organization has been intensively studied in recent years. Therefore, information about the BCR structure provided novel insights into molecular mechanisms of BCR signal initiation and signal transduction. In 2022, two independent groups successfully identified the human BCR structure using cryo-electron microscopy [59,60]. Furthermore, the identification of the murine BCR structure supplemented the data on human BCR [61].

Each of the approximately 120,000 BCR receptor molecules present at the surface of a mature B cell contains a membrane-embedded Ig (class M, D, G, A, or E) composed of two light and two heavy chains linked with disulfide bonds [61,62]. There is a strong evidence that the immunoglobulin-CD79a/CD79b stoichiometric ratio is 1:1, supporting the attachment of CD79a/CD79b to the immunoglobulin (in a non-covalent manner) [59,60,61,63,64]. The membrane attachment of the immunoglobulin molecule is mediated via C-terminal regions of Ig heavy chains, specifically by their transmembrane domains [65]. Multiple studies indicated that the cytoplasmic tails of membrane-bound immunoglobulins mediate isotype-specific BCR signaling. The immunoglobulin tail tyrosine (ITT) signaling motif was described in membrane-bound IgG and seems to mediate the reactivation of IgG-switched memory B cells. Following antigen-induced SYK (spleen tyrosine kinase)-mediated phosphorylation, the ITT facilitates docking of adaptor protein Grb2 (growth factor receptor-bound protein 2). Grb2 in turn recruits BTK, which leads to the amplification of Ca^2+^ mobilization and resulting amplification of BCR signaling [66,67,68,69]. However, the CD79a/CD79b heterodimer is critically important for a general BCR signal transduction [70,71]. CD79a/CD79b heterodimeric unit contains three domains (extracellular, transmembrane, and intracellular) and is bound together with disulfide bonds formed between cysteine residues of its extracellular domains [72]. Interestingly, CD79a and CD79b extracellular domains are the least conserved regions of CD79a and CD79b molecules with significant interspecies variability. Experiments in mouse B cells showed that their replacement with the human counterpart does not affect BCR assembly and signaling [73].

Interactions between Ig and CD79a/CD79b complex result in the formation of four-helix transmembrane bundle whose conserved nature assures correct assembly of any BCR isotype [60,74]. These tight interactions within the cell membrane are crucial for the assembly, stability and functionality of BCR, suggesting co-folding of BCR components during BCR complex formation [63]. Moreover, recent study demonstrated that the assembly and signaling features of BCR additionally depend on protein–protein interactions between transmembrane domains of CD79a and CD79b [72].

Specific interactions between the membrane-bound immunoglobulin and CD79a/CD79b are dependent on the BCR isotype. The IgM immunoglobulin binds to the CD79a/CD79b heterodimer extracellular domain from the side, whereas the IgG immunoglobulin interacts with the top of the CD79a/CD79b complex. It further suggests isotype specific differences in BCR signaling [74,75].

Intracellular domains of CD79a and CD79b have an immunoreceptor tyrosine-based activation motif (ITAM) made up of 26 amino acid residues and containing two critical tyrosine residues. ITAM tyrosines can undergo phosphorylation by the Src-family of kinases and are directly involved in BCR signal mediation [17,76,77]. Furthermore, it seems that ITAM phosphorylation also contributes to the regulation of BCR internalization [78].

**Figure 1:**
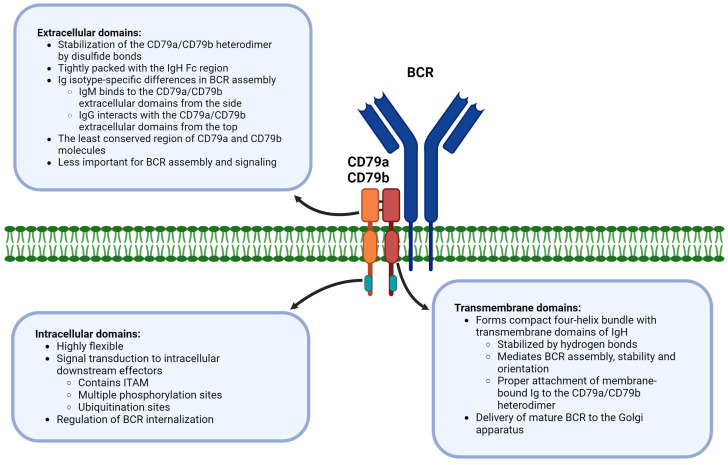
Distinct function of CD79a/CD79b domains in B-cell receptor signaling. Abbreviations: BCR—B-cell receptor; Fc region—fragment crystallizable region; IgH—immunoglobulin heavy chain; ITAM—immunoreceptor tyrosine-based activation motif. This figure was created with biorender.com.

The in-depth analysis of the cryo-electron microscopy BCR structure and additional studies of BCR functionality revealed that individual CD79a and CD79b domains mediate distinct processes to coordinate BCR signaling (Figure 1).

## 5. The CD79a/CD79b Heterodimer Is Critically Important for BCR Functionality

BCR plays a pivotal role in B cell biology. It regulates antigen uptake, antigen presentation, cell survival, proliferation, activation, differentiation, metabolism, B cell negative selection, cellular death, and B cell anergy [1,11,17,19,28,79,80,81,82,83]. Results of multiple studies and our current understanding of BCR signaling suggest that the CD79a/CD79b heterodimer is among key decisive regulators of BCR signaling and B cell fate.

CD79a and CD79b were implicated in the regulation of membrane-bound IgM expression and BCR complex formation and stability. The functional CD79a/CD79b complex promotes IgM transport and increases its surface levels via adjusting its glycosylation [2]. Furthermore, CD79a and CD79b facilitate assembly and steadiness of BCR by stabilizing each other to form the CD79a/CD79b dimer, mediate glycosylation of each other and IgM, as well as deliver the assembled receptor to the Golgi apparatus [2,84]. CD79a- and CD79b-mediated intracellular trafficking has been suggested to depend on their transmembrane domains’ interactions and ubiquitination patterns [2,78,85].

Two of the main pathways mediating survival signals downstream of BCR are NF-κB signaling (e.g., anti-apoptotic genes upregulation) and PI3K/AKT signaling (e.g., via its downstream effector transcriptional factor FOXO1) [86,87,88,89]. In naïve B cells, both CD79a and CD79b are required to physically interact with B-cell activating factor receptor (BAFFR), whose ligand, the B-cell activating factor (BAFF), initiates pro-survival PI3K/AKT signaling upon binding to BAFFR [90]. BCR signaling, combined with co-stimulation from CD40, BAFFR, and TLRs, regulates antibody-mediated immune responses requiring class switch recombination [21]. Interestingly, He et al. reported that association of CD19 with CD79b alone could trigger an alternative pathway to promote survival, fitness, and growth of B cells in a PI3K-dependent fashion [76]. Antigenic BCR stimulation and consequent signaling events also induce association of CD79a/CD79b heterodimers with MHC class II molecules. CD79a/CR79b heterodimers thus contribute to signal transduction following MHC class II activation (engaging Src-family tyrosine kinases and Ca^2+^ signal) [91,92]. The indirect mechanism by which the CD79a/CD79b heterodimer promotes cell survival is the already mentioned enhancement of IgM surface expression [2]. BCR signaling could also induce autophagy, which is involved in the regulation of cell survival and metabolic homeostasis of B cells [28,93]. Furthermore, B cell polarization and BCR intracellular trafficking are regulated by autophagy-related proteins mobilized upon BCR signaling activation [94]. However, the exact role of CD79a and CD79b in these signaling events, as well as details about their interaction with other BCR co-receptors, are not yet fully known and may not be limited to a simple recruitment of BCR signaling mediators. Importantly, autophagy-related regulation of BCR trafficking may complement the already reported effects of CD79a and/or CD79b phosphorylation and ubiquitination on antigen processing and presentation [78,85]. As mentioned above, proliferation and activation of B cells is supported by BCR-mediated PI3K signaling activation and consequent stimulation of mitochondrial biogenesis and elevated glucose uptake [26,28,95]. Nevertheless, it is important to emphasize that while BCR signaling is important for metabolic reprogramming of B cells, the ITAM-containing CD79a and CD79b BCR components are not indispensable for it [96].

The critical importance of CD79a and CD79b BCR subunits for BCR functionality, signal transmission, and signal regulation was largely established by studies of CD79a and CD79b posttranslational modifications.

## 6. BCR Signaling Is Regulated by Phosphorylation, Ubiquitination, and Glycosylation of CD79a and CD79b

**Figure 2:**
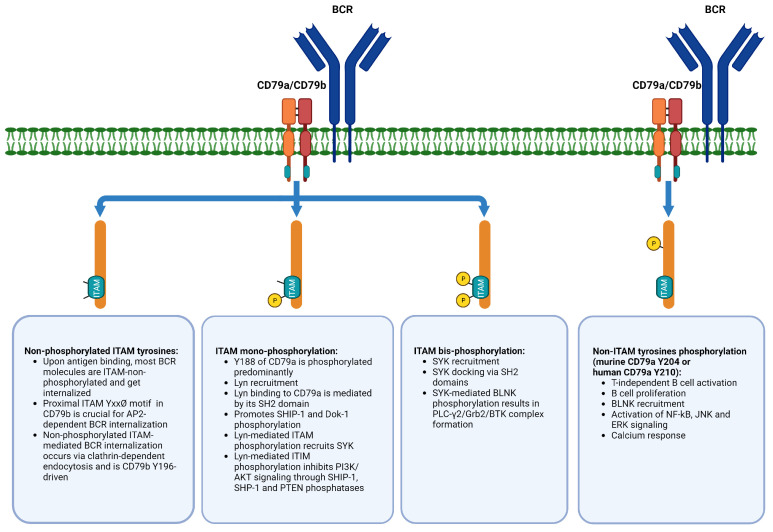
Phosphorylation of ITAM and non-ITAM tyrosines of CD79a and CD79b differentially modulates BCR signaling. Abbreviations: BLNK—B-cell linker protein; BCR—B-cell receptor; BTK—Bruton’s tyrosine kinase; Dok-1—docking protein 1; ERK—extracellular signal-regulated kinase; Grb2—growth factor receptor-bound protein 2; ITAM—immunoreceptor tyrosine-based activation motif; ITIM—immunoreceptor tyrosine-based inhibitory motif; JNK—c-Jun N-terminal kinase; Lyn—Lck/Yes novel tyrosine kinase; NF-κB—nuclear factor kappa-light-chain-enhancer of activated B cells; P—phosphate group; PI3K—phosphoinositide 3-kinase; PLC-γ2—phosphoinositide phospholipase C-γ-2; PTEN—phosphatase and tensin homolog; SHIP-1—Src homology 2 (SH2) domain-containing inositol-5-phosphatase 1; SHP-1—Src homology region 2 domain-containing phosphatase-1; SYK—spleen-associated tyrosine kinase. This figure was created with biorender.com.

ITAM phosphorylation is critical for signal transduction in ITAM-containing receptors. ITAM motifs are relatively common across various receptors in different immune cells including, e.g., T cells, NK cells, macrophages, or dendritic cells [97]. It is generally accepted that phosphorylation of both ITAM tyrosine residues by Src-family tyrosine kinases such as Lyn, Fyn, or Blk recruits SYK and the resulting complex activates downstream signaling pathways (e.g., PI3K/AKT, NF-κB, and MAPK) [17,38,98,99]. Furthermore, recent studies suggested that phosphorylation patterns of CD79a and CD79b ITAMs might affect BCR cellular localization and determine which downstream kinase is recruited to modulate BCR signaling (Figure 2).

Upon antigen binding, most cell surface BCR molecules are internalized for antigen processing and further presentation [5,100]. This process can occur even when ITAM tyrosine residues remain non-phosphorylated [78,101]. Importantly, non-phosphorylated ITAM-mediated BCR endocytosis is critically dependent on mCD79b Y195, but not mCD79a Y182 (murine CD79a, these tyrosine residues correspond to CD79b Y196 and CD79a Y188 in humans, respectively) and relies on adaptor protein 2 (AP2, an important mediator molecule involved in clathrin-mediated endocytosis). Phosphorylation of ITAM tyrosine residues prevents AP2 binding to the BCR YxxØ endocytosis motif and hence BCR internalization [101].

Results of multiple studies indicate that mono- or bis-phosphorylation of ITAM tyrosine residues determines whether Lyn or SYK is recruited. Lyn is recruited upon ITAM mono-phosphorylation, while SYK docking occurs in response to ITAM bis-phosphorylation (observed only in approximately 20% of activated BCR molecules) [102,103,104]. It has been also reported that Src-family kinases are preferentially recognized by CD79a in contrast to CD79b [105]. Comparing the two CD79a ITAM phosphorylation sites, mCD79a Y182 mediates Lyn recruitment in comparison to mCD79a Y193 (murine CD79a, these tyrosine residues correspond to Y188 and Y199 in human CD79a, respectively) [104]. Lyn acts as a molecular switch in BCR signaling, working in a bidirectional manner. Like other Src-family kinases, it phosphorylates CD79a/CD79b ITAMs to provide SH2 domain-associated SYK docking. On the other hand, Lyn phosphorylates ITIMs (immunoreceptor tyrosine-based inhibitory motifs) in ITIM containing receptors with consequent recruitment of phosphatases such as SHIP-1 (Src homology region 2 domain-containing inositol 5′ phosphatase 1), SHP-1 (Src homology region 2 domain-containing tyrosine phosphatase), and PTEN (phosphatase and tensin homolog) to downregulate BCR signaling [102,106,107]. Lyn-mediated SYK recruitment activates the B-cell linker (BLNK) protein, which promotes assembly of phosphoinositide phospholipase C-γ-2 (PLC-γ2), BTK, and adaptor protein Grb2 into a multimolecular regulatory complex leading to downstream NF-κB and PI3K/AKT signaling activation [17]. However, within components of this classical BCR signalosome, Lyn is not indispensable. Lyn deficiency in Lyn knock out (KO) models can be compensated by other members of Src-family kinases [108,109]. At the same time, Lyn is critical for signalosome-independent IL-4-mediated alternative pathway of BCR signaling activation [108].

The above-mentioned Lyn-dependent negative regulation of BCR signaling relies on SHIP-1- and PTEN-catalyzed dephosphorylation of phosphatidylinositol (3,4,5)-trisphosphate (PtdIns(3,4,5)P3), blocking the PI3K/AKT pathway [103]. Moreover, Lyn-mediated SHP-1 activation leads to CD79a/CD79b ITAMs and SYK dephosphorylation, forming a negative feedback loop [110,111]. Therefore, Lyn determines the signal transduction strength, mediating the balance between phosphorylation/dephosphorylation of key regulatory intracellular BCR signaling components. Therefore, it affects B cell fate decisions. Lyn recruitment, which depends on phosphorylation patterns of CD79a/CD79b, upregulates intracellular Ca^2+^ signaling through SYK-mediated PLC-γ2 activation and downregulates it via PtdIns(3,4,5)P3 degradation (mediated by SHIP-1 and PTEN). It was reported that quantitatively distinct Ca^2+^ signaling patterns downstream of BCR activation regulate B cell survival by NF-κB engagement and B cell proliferation via NFAT, mTORC1, and c-Myc activation [18].

Experimental evidence indicates that other than classical ITAM tyrosines of CD79a (amino acid positions 188 and 199) are also substrates of phosphorylation and are involved in regulation of BCR signaling. mCD79a Y176 and Y204 are required for BLNK recruitment (a key adaptor phosphorylated by SYK). mCD79a Y204 phosphorylation is of particular importance for BLNK recruitment. It places SYK and BLNK in a close proximity to allow further BLNK activation. It also links SYK with downstream BCR signaling events including Ca^2+^ mobilization, NF-κB, ERK, and JNK activation [112,113,114]. The murine mCD79a Y176 corresponds to human CD79a Y182 (a third tyrosine within the ITAM domain) and murine mCD79a Y204 corresponds to human CD79a Y210 (a non-ITAM downstream tyrosine). Therefore, both ITAM and non-ITAM tyrosine residues are subject to phosphorylation and are all crucial for BCR signal transduction and regulation of its strength. The strength of BCR signaling also depends on intricate equilibrium between kinases and phosphatases.

Phosphorylation is certainly a key covalent post-translational modification of CD79a/CD79b which regulates BCR signaling. However, several studies suggested that both CD79a and CD79b lysine sites are utilized for ubiquitin attachment and are therefore prone to ubiquitination [78,85,115,116,117,118]. As mentioned previously (Figure 1), ubiquitination sites are located in the intracellular domain of CD79a and CD79b [115]. Upon BCR-antigen binding, CD79a and CD79b are subject to rapid ubiquitination (within 5 min) [117]. Time-dependent kinetics of CD79a and CD79b ubiquitination and its role in BCR receptor function still needs to be elucidated, but it has been demonstrated that CD79b is ubiquitinated first, followed by a CD79a ubiquitination [85].

Ubiquitination might have a much wider significance in proximal BCR signaling regulation in general since CD79a and CD79b are ubiquitinated concurrently with Lyn and SYK. Importantly, CD79a phosphorylation (but not CD79b phosphorylation) seems to promote CD79a ubiquitination. Moreover, concurrent activation of SYK employs ubiquitin ligases such as c-Cbl and Cbl-b, which may further recruit Itch (another ligase involved in ubiquitination-dependent BCR signal regulation) [117,119]. Involvement of ubiquitination at early stages of BCR signaling suggests that it may be a versatile signaling mark. Several studies showed that CD79a and/or CD79b ubiquitination specifically targets BCR for endocytosis even if ITAM tyrosines are phosphorylated and that it affects intracellular trafficking, endosome signaling, and antigen processing and presentation [78,85,116,118]. Veselits et al. reported that CD79b ubiquitination is necessary for PI3K activation and PIP_3_ accumulation in BCR-containing endosomes, affecting consequent sorting into the major histocompatibility complex (MHC) class II antigen-presenting compartment and endocytic trafficking [116]. Moreover, KLHL14 (Kelch-Like Family Member 14, a tumor-suppressing chaperone regulating protein folding, frequently mutated in lymphomas) promotes CD79a and CD79b ubiquitination and their consequent downregulation [115]. Since CD79a/CD79b ubiquitination is involved in many aspects of BCR signaling, it could be potentially targetable; however, the exact mechanisms of CD79a/CD79b ubiquitination and its regulation are not fully clarified.

Recent studies also showed that CD79a/CD79b could be N-glycosylated and have linked this modification to the regulation of BCR surface expression. Abnormal glycosylation of CD79a results in retention of BCR complex in the endoplasmic reticulum for further proper folding and assembly, leading to a reduction in its translocation to the cell membrane [84]. Apart from the above-mentioned CD79a/CD79b ubiquitination, KLHL14 protein affects also CD79a/CD79b glycosylation. It regulates immature glycosylated BCR stability and turnover in the endoplasmic reticulum [115].

In summary, post-translational modifications of CD79a/CD79b heterodimer define its own assembly, BCR expression, BCR endocytosis, strength of BCR signaling, intracellular trafficking, and antigen processing and presentation.

## 7. BCR Signaling Is Regulated at the Level of CD79a/CD79b Heterodimer by Physical Interactions with Regulatory Molecules

**Figure 3:**
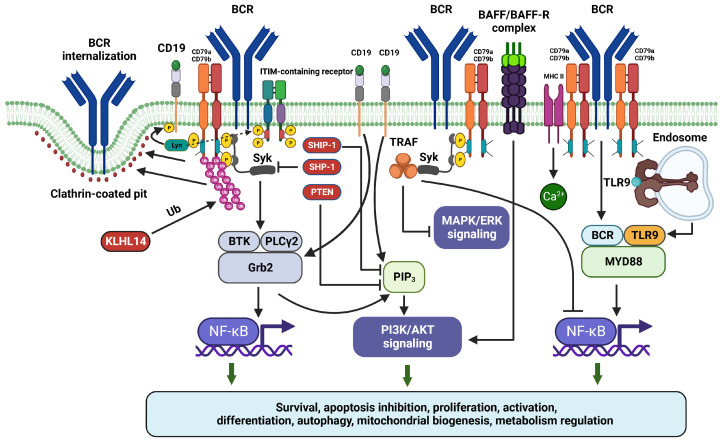
The role of CD79a/CD79b in B-cell receptor signaling in normal B cells. Abbreviations: BAFF—B-cell activating factor; BAFFR—B-cell activating factor receptor; BCR—B-cell receptor; BTK—Bruton’s tyrosine kinase; ERK—extracellular signal-regulated kinase; Grb2—growth factor receptor-bound protein 2; ITAM—immunoreceptor tyrosine-based activation motif; ITIM—immunoreceptor tyrosine-based inhibitory motif; KLHL14—Kelch Like Family Member 14; Lyn—Lck/Yes novel tyrosine kinase; MAPK—mitogen-activated protein kinase; MHC II—major histocompatibility complex class II; NF-κB—nuclear factor kappa-light-chain-enhancer of activated B cells; P—phosphate group; PI3K—phosphoinositide 3-kinase; PIP3—phosphatidylinositol (3,4,5)-trisphosphate; PLCγ2—phosphoinositide phospholipase C-γ-2; PTEN—phosphatase and tensin homolog; SHIP-1—Src homology region 2 domain-containing inositol 5′ phosphatase 1; SHP-1—Src homology region 2 domain-containing tyrosine phosphatase; Syk—spleen-associated tyrosine kinase; TLR9—toll-like receptor 9; TRAF3—tumor necrosis factor receptor (TNFR)-associated factor 3; Ub—ubiquitination. This figure was created with biorender.com.

As already mentioned above, CD79a/CD79b heterodimer or its components can physically interact with a wide range of regulators to modulate BCR signaling (summarized in Figure 3). Classical BCR signaling requires direct Lyn recruitment or SYK docking to promote further activation of downstream kinases [102,103,104]. On the other hand, multiple studies have recently demonstrated that CD79a and CD79b might have substantially larger interactomes.

As mentioned above, CD79a/CD79b heterodimeric unit determines BCR internalization via its ubiquitination and ITAM phosphorylation. Mono-phosphorylation of CD79 ITAMs recruits Lyn, while bis-phosphorylation docks SYK with further activation of BTK and PLCγ2 and resulting downstream activation of NF-κB and PI3K/AKT signaling. CD79a/CD79b unit mediates also co-signaling from CD19, BAFFR, MHC II, and TLR9 and is regulated by KLHL14 and TRAF3 (tumor necrosis factor receptor (TNFR)-associated factor 3). CD79b seems to localize in a close proximity to CD19 even when it is not associated with other components of BCR (i.e., Ig and CD79a). This interaction helps to maintain pro-survival signal in B cells in an ITAM/PI3K-dependent manner [76]. PI3K recruitment upon stimulation of CD19 co-receptor requires its Lyn-dependent phosphorylation [120]. Direct interactions of CD79a and CD79b with TLR9 lead to MYD88 recruitment and hence NF-κB signaling upregulation via the MYD88-TLR9-BCR supercomplex [49]. In addition to NF-κB signaling, ligand binding to BAFFR (which could be either attached or located closely to CD79a and CD79b) upregulates also PI3K/AKT as another critical BCR downstream pathway [90]. Furthermore, CD79b, along with other proteins involved in proximal BCR signaling (SYK and BTK), interacts with TRAF3 (a negative regulator of BCR signaling). Therefore, this interaction results in reduction of non-canonical NF-κB and MAPK/ERK pathway activation without affecting canonical NF-κB and PI3K/AKT signal [121]. Notably, TRAF3 has been well established as a regulator of CD40 and BAFFR signaling in B cells [122,123]. The above-mentioned KLHL14 is another negative regulator of BCR signaling which physically interacts with CD79a and CD79b [115]. As mentioned above, CD79a/CR79b heterodimers could also associate with MHC class II and contribute to its downstream signal transduction [91,92].

Expansion of CD79a and CD79b molecular interaction information further supports the critical role of these BCR components in a complex regulation of downstream BCR signaling. It can be assumed that CD79a/CD79b unit, especially its CD79b component, may represent a signaling hub mediating crosstalk between BCR and its co-receptors.

## 8. CD79a and CD79b Are Important Regulators of Proximal and Distal BCR Signaling in Malignant B Cells

Availability of next-generation sequencing has significantly expanded our understanding of mutational profiles in B-cell derived malignancies, increased the number of known tumor driver genes, and provided information about additional mechanisms of tumorigenesis. Tumorigenic contribution of *CD79A* and *CD79B* mutations is well studied and could be demonstrated in NHL as an example. Mutation frequency of *CD79B* varies between 4 and 23%, largely depending on NHL type [54,57,124,125]. Mutations of *CD79A* are present in approximately 3–4% of NHL [57,58]. *CD79A* and *CD79B* mutations most frequently affect ITAM regions. In general, *CD79A* alterations primarily result in removal of the entire ITAM region, while *CD79B* mutations frequently affect the first tyrosine residue of ITAM, the Y196 [41,126,127]. The main example of uneven distribution of *CD79A* and *CD79B* mutations between lymphoma subtypes is DLBCL. *CD79A* and *CD79B* mutations are observed in up to 30% of ABC DLBCL cases and only in 3% of GCB DLBCL tumors [41,48,57,126,128,129]. None of the currently proposed genetic clustering algorithms uses *CD79A* mutations as a genetic feature of any DLBCL genetic subtype. However, frequency of *CD79A* alterations is higher in EZB cluster [54,55,56,57,58,130]. In contrast to *CD79A*, *CD79B* mutations are one of the most enriched genetic features of MCD cluster and are very frequent in BN2 and A53 clusters in the GenClass and LymphGen algorithm-based genetic classifications, respectively [56]. According to the alternative genetic DLBCL classifications, *CD79B* mutations are enriched in Cluster 5, MYD88, and MCD^2-S^ genetic subtypes, which are all MCD cluster equivalents [54,55,58]. This genetic DLBCL subtype (with high frequency of *CD79B* mutations) is robust and omnipresent across all genomic classifications of DLBCL and is strongly associated with active BCR signaling. In lymphoma, *CD79B* mutations augment BCR surface levels via reduction of BCR endocytosis, preventing BCR binding to clathrin-coated pits [3,41,48,101,126,131]. Importantly, BCR signaling strength seems to be proportional to CD79b expression levels in NHL [132]. Another widely recognized mechanism through which mutated CD79b increases BCR signaling is its inability to properly activate Lyn, a Src-family tyrosine kinase triggering negative feedback loop-based inhibition of BCR signaling [3,41,126,128,131]. Reduced Lyn activity also contributes to overcoming B cell anergy [41]. Importantly, CD79b with mutated ITAMs promotes BCR clustering in ABC DLBCL, which is a characteristic consequence of BCR-antigen binding [3,38,50,133]. It indirectly supports the important role of CD79b protein alterations in chronic active BCR signaling augmentation. Therefore, CD79b mutations might promote pro-survival active BCR signaling via BCR surface levels upregulation, enhancement of BCR clustering, inhibition of BCR internalization, and reduced BCR signaling negative regulation. On top of ITAM tyrosines mutations, Andrades et al. reported that *CD79B* gene is frequently affected by recurrent splice site mutations in DLBCL. These mutations frequently lead to intron 4 retention with premature termination of CD79b translation. It also results in BCR overexpression and enhancement of NF-κB and AKT signaling [134]. CD79a and CD79b molecules (and possibly their mutations) further affect the CD79b-CD19 and BCR-BAFFR mediated PI3K/AKT activation and BCR/TLR9/MYD88 and CARD11/BCL10/MALT1 complexes formation with consequent NF-κB signaling activation.

*CD79A* mutations are much less frequent and were not identified within any genetic DLBCL cluster. On the other hand, it was reported that *CD79A* mutations can contribute to activation and enhancement of chronic active BCR signaling in ABC DLBCL as well [135]. Importantly, wt CD79a, specifically its ITAM Y188 and its phosphorylation, are critical for tonic BCR signal mediation in GCB DLBCL [50].

Frequencies and signaling consequences of *CD79A* and *CD79B* mutations are overviewed in Table 1 and Table 2, respectively. Most genetic studies focused only on ITAM regions, therefore, data on frequencies of non-ITAM region mutations are limited. However, Lohr et al. reported that in DLBLC, 12.5% of *CD79B* mutations affect non-ITAM regions [136]. High rate of mutations in extracellular and transmembrane domains of CD79b (over 30% of patients) was detected also in CLL [137]. Therefore, it would be of great interest to analyze specifically frequency and eventual signaling consequences of mutations affecting non-ITAM regions across various B-cell derived malignancies.

**Figure 4:**
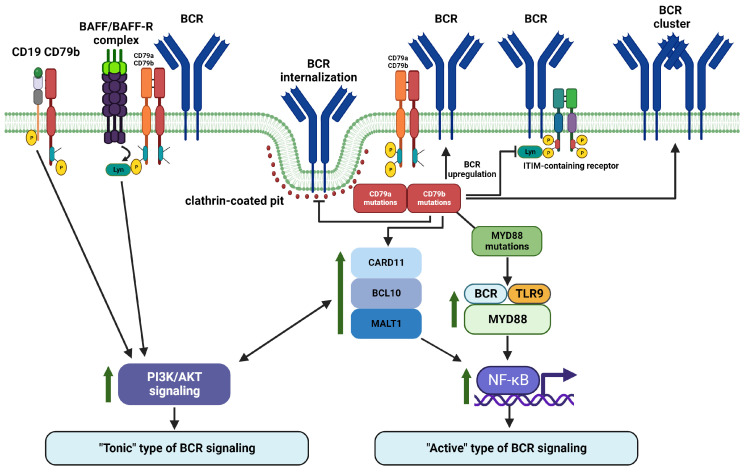
Contribution of CD79a and CD79b to the BCR-related signaling in lymphomas as a component of the cancer-mutation network. Abbreviations: BAFF—B-cell activating factor; BAFFR—B-cell activating factor receptor; BCL10—B-cell lymphoma 10; BCR—B-cell receptor; CARD11—caspase recruitment domain family member 11; DLBCL—diffuse large B-cell lymphoma; ITAM—immunoreceptor tyrosine-based activation motif; ITIM—immunoreceptor tyrosine-based inhibitory motif; Lyn—Lck/Yes novel tyrosine kinase; MALT1—mucosa-associated lymphoid tissue lymphoma translocation protein 1; MYD88—myeloid differentiation primary response 88; NF-κB—nuclear factor kappa-light-chain-enhancer of activated B cells; PI3K—phosphoinositide 3-kinase; P—phosphate group; TLR9—toll-like receptor 9. This figure was created with biorender.com.

Taken together, it could be hypothesized that chronic active BCR signaling in ABC DLBCL relies primarily on CD79b, while CD79a contributes more to tonic BCR signaling in GCB DLBCL. However, more studies are necessary to elucidate the exact contribution of either CD79a or CD79b (and their mutations) to various types and states of BCR signaling in lymphoma. An overview of CD79a/CD79b involvement in lymphoma associated BCR signaling is provided in Figure 4.

Importantly, ABC DLBCL tumor cell survival is critically dependent on BCR triggered NF-κB activation [50,154,155,156]. BCR downstream NF-κB activation is mediated by CD79a and CD79b and is enhanced by their mutations. However, within the pathologically active BCR signaling in lymphoma, *CD79A* or *CD79B* mutations cannot be considered separately from other mutations. It is only one molecular event within a quite complex BCR signaling and NF-κB activation process. As an example of this complexity, *CD79A* and *CD79B* mutations were included in a large network of 153 lymphoma-altered genes related to NF-κB signaling [124]. Other studies also reported that *CD79A* and *CD79B* mutations modify distal BCR signaling in collaboration with other mutations of the BCR signaling network via modulation of NF-κB signaling (with an effect on survival, apoptosis, and proliferation of tumor cells). Mutated CD79a or CD79b proteins cannot trigger BCR signaling by itself; they “only” amplify the signal and modulates its strength and direction [135]. This relationship was described specifically for *CARD11* (caspase recruitment domain family member 11) mutations in ABC DLBCL. CARD11 acts as a component of the CARD11-BCL10-MALT1 signalosome complex involved in bridging proximal and distal BCR signaling towards NF-κB activation [157,158]. *CARD11* mutations seem to be necessary for mutated CD79a/CD79b-mediated NF-κB activation [48]. In addition, viability of mutated *CD79B* ABC DLBCL cells critically relies on BTK that fuels canonical NF-κB signaling via regulation of downstream mucosa-associated lymphoid tissue lymphoma translocation protein 1 (MALT1) [129]. *CD79B* mutations not only sustain anti-apoptotic and proliferation-inducing NF-κB signaling directly but also support the interplay between other BCR-associated signaling pathways to ensure the survival of ABC DLBCL cells. It was reported that in two model ABC DLBCL cell lines with mutated *CD79B*, PI3K signaling and downstream PDK1 (putative 3-phosphoinositide-dependent kinase 1) activation are essential events for CARD11-BCL10-MALT1 signalosome complex-mediated NF-κB activation [159]. This suggests a substantial interaction between PI3K signaling and the NF-κB pathway in *CD79B*-mutated ABC DLBCL. In MCL, pro-survival PI3K/AKT/mTOR signaling is sustained by wt CD79a and requires activity of Lyn [160]. This is consistent with the role of CD79a in promoting pro-survival PI3K/AKT/mTOR signaling in GCB DLBCL [50]. In contrast to ABC DLBCL, GCB DLBCL cells rely on the PI3K/AKT/mTOR to support their survival [161,162]. Therefore, GCB DLBCL is characterized by tonic BCR signaling where CD79a phosphorylation results in activation of downstream PI3K/AKT pathway [50].

In lymphomas, *CD79B* mutations also commonly co-occur with *MYD88* mutations, especially *MYD88^L265P^*. *MYD88* mutations have higher prevalence in cases with extranodal localization and mediate active BCR signaling phenotype within the MCD genotype of DLBCL [3,56,58,126,127,130,163,164,165]. Multiple studies reported a combined biological effect of *CD79B* and *MYD88* mutations. Importantly, none of these two mutated genes can induce malignant transformation alone as they need to act in collaboration with other driver mutations [127,166]. It has been shown that only the combination of *CD79B* and *MYD88* mutations prevented anergy of autoantigen-stimulated B cells and allowed their plasmablastic differentiation [152]. The most common *MYD88^L265P^* mutation results in overactivation of MYD88 protein, a molecular adaptor that normally promotes NF-κB signaling via IRAK (IL-1 receptor-associated kinase) recruitment and formation of the so-called myddosome complex [167]. Myddosomes are large oligomeric signaling complexes that are assembled within TLRs and IL-1 signaling. Myddosomes are primarily formed by MYD88 and IRAKs and act as a scaffold for further downstream signal transduction [168,169,170,171]. Phelan et al. demonstrated that *CD79B*-*MYD88* mutations simultaneously facilitate intermolecular interactions to form a three-component MYD88-TLR9-BCR (My-T-BCR) supercomplex, which drives NF-κB and mTOR signaling activation [49]. Furthermore, lymphoma sequencing studies revealed that the network of concurrently mutated genes in MCD DLBCL cluster includes inactivating mutations of *KLHL14*, which promotes My-T-BCR-dependent NF-κB signaling via reduction of CD79a and CD79b ubiquitination [115]. It is worth mentioning that enhancement of CD79b-mediated non-canonical NF-κB signaling has been associated with TRAF3 insufficiency in lymphoma [121].

In addition to the My-T-BCR supercomplex, CD79a/CD79b maintains pro-survival PI3K/AKT signaling via interaction with BCR co-receptors BAFFR and CD19 in B-cell NHL [76,90]. The pro-survival signal comes from BAFF-dependent Lyn-mediated activation of PI3K/AKT [90]. A similar PI3K-dependent pro-survival signal is sustained by CD79b through CD19 activation. It is ITAM-dependent, can occur even in the absence of other BCR components (IgM and CD79a), and is considered an alternative lymphoma survival supporting mechanism [76].

Taken together, CD79a and CD79b BCR components could be considered critical regulators of altered signal flow in the complex network of deregulated BCR signaling in NHL. This suggests that CD79a/CD79b complex might represent a suitable and universal therapeutical target for B-cell malignancies and possibly other B-cell-related disorders. On the other hand, BCR signaling deregulation occurs in response to multiple mutations in several interacting and BCR regulatory elements adjusting pro-survival BCR signaling in a relatively diverse manner.

## 9. Open Questions and Future Directions

Next-generation sequencing-based subtyping of DLBCL has improved the accuracy of disease outcome prediction. Particularly, concomitant *CD79B* and *MYD88* mutations (characteristic for the MCD class of DLBCL) are associated with worse prognosis [3,55,57,126,172,173]. Importantly, a recent DLBCL meta-analysis showed that *CD79B* mutations had higher predictive value for disease progression and treatment outcomes than *MYD88* mutations [164]. Inferior survival of patients with *CD79B* mutations might be related to a specific disease biology and associated with an increased risk of chemo-refractory disease and relapse [54]. Indeed, transcriptomic, and proteomic studies showed downregulation of CD79b in refractory DLBCL. This suggests that CD79b targeted therapy, such as polatuzumab vedotin (anti-CD79b monomethyl auristatin E antibody-drug conjugate), might not be best suited in this clinical situation [174]. On the other hand, overexpression of CD79b in ABC DLBCL contributes to ibrutinib (a potent BTK inhibitor) resistance. Similarly, MCL tumors with higher baseline levels of CD79b expression required higher concentrations of ibrutinib for efficient BCR signal suppression [132,175,176]. None of these correlations were observed for CD79a upregulation. On the other hand, it was reported that BTK inhibition increases CD79a phosphorylation as a part of compensation feedback loop. It leads to activation of multiple key BCR signal mediators with consequent proximal BCR signal rewiring [177]. Increased CD79a/CD79b activity might be one of general resistance mechanisms. It was reported that CD79a-mediated Lyn-dependent activation of PI3K/AKT/mTOR signaling also occurs in bortezomib (an FDA-approved 26S proteasome inhibitor) resistant MCL [160]. Future studies regarding CD79b activation and interactions in malignant B cells are necessary to fully understand its contribution to drug resistance and to confirm its eventual diagnostic significance as a reliable resistance biomarker. Pre-clinical in vitro-based studies could provide general information how CD79a and CD79b altered expression, mutations, and/or phosphorylation patterns affect sensitivity to various inhibitors. The variability of CD79b expression levels between different types of NHLs and individual tumors (which likely predicts BCR activity) indicates that CD79b expression could be evaluated as a possible biomarker for effective BCR inhibition. The evaluation of CD79a and CD79b pre-treatment expression levels (as well as its treatment resistance-related changes) should be implemented in clinical trials with BCR signaling inhibitors. Moreover, CD79b assessment could be considered in studies of other targeted therapies where BCR signaling activation might contribute to treatment resistance. Explanation of CD79a/CD79b signaling details, as outlined below, might help to identify additional therapeutic options for personalized B-cell derived malignancies treatment.

BCR signaling, as well as the functional involvement of CD79a and CD79b molecules, has already been extensively studied; however, many important issues remain to be addressed. What are the underlying transcription factors, pathways, or other mechanisms regulating CD79a and CD79b expression? Implementation of genome-wide KO screen approaches in model lymphoma cell lines might help to identify these factors. It could also explain differences in CD79a and CD79b expression levels (in normal as well as malignant B cells) and lead to the identification of novel approaches for BCR signaling inhibition.

Multiple questions also remain to be answered in relation to CD79a/CD79b ITAM phosphorylation. How is the ITAM phosphorylation regulated? Do CD79a/CD79b conformational changes mediate CD79a/CD79b ITAM phosphorylation? What are the exact and detailed characteristics of CD79a/CD79b interactions with Src-family kinases? Src-family tyrosine kinases generally have a BCR signaling activation role; however, Lyn forms a negative feedback loop highlighting its unique and important role within BCR signal regulation. The complexity, interaction, activation/inhibition balance, and co-regulation between CD79a/CD79b and Src-family kinases are not fully clarified. Moreover, are there any other kinases and phosphatases involved in ITAM phosphorylation and dephosphorylation? Basic molecular biology studies using targeted KO, protein–protein interaction assessment, or structural biology methods might bring such information.

Further studies should also focus on mechanisms of CD79a and CD79b ubiquitination and glycosylation as another layer of BCR signal regulation. Similarly, it is important to identify how is BCR signaling affected by tumor-related disruption of redox homeostasis and reactive oxygen species (ROS) production [178,179]. Novel proteomics-based studies of the CD79a/CD79b interactome might provide additional information on these unresolved issues.

Given the heterogenous nature of B-cell derived malignancies, it is similarly important to identify mechanisms by which *CD79A* and *CD79B* mutations modulate BCR signaling balance towards a particular effector pathway or pathways in a complex landscape of multiple co-occurring driver mutations. The direct involvement of CD79a/CD79b in CD19, BAFFR and TLR9 signaling raises a question of their possible critical role in the formation of a mutual BCR-centered signaling network [49,76,90,180]. Is the CD79a/CD79b heterodimer a signaling hub coordinating signals from various BCR co-receptors? Interpretation of genomic data and experimental studies also suggest that CD79a and CD79b signal mediation might be substantially different between normal and malignant B cells.

## 10. Conclusions

CD79a and CD79b molecules are at the center of a very complex BCR signaling network with critical functional implications for normal as well as malignant B cells. Moreover, CD79a/CD79b unit mediates signaling crosstalk between BCR and its co-receptors, which supports its role as an important B cell signaling hub. Solving above outlined open issues could further expand our understanding of BCR signal initiation and propagation and allow us to further specify the complexity of CD79a/CD79b signaling and possibly identify novel therapeutic targets for B-cell related disorders.

## Figures and Tables

**Table 1:** Frequency of *CD79A* mutations and related B-cell receptor signaling consequences.

Frequency of Cases with Any *CD79A* ITAM Mutation	*CD79A* Mutation Subtypes	Frequency of Cases with *CD79A* Mutation Subtypes *	Signaling Consequences of *CD79A* Mutation Subtypes
6% in DLBCL [124] 5% in unclassified DLBCL [125] 2.5% in CD5^+^ DLBCL [138] 0.7% in DLBCL [58] 6.5% in ABC DLBCL [139] 2.9% in ABC DLBCL [48] 0% in ABC DLBCL [125] 0% in GCB DLBCL [139] 6.3% in PCLBCL-LT [131] 5.5% in WM [140] 3% in FL [141] 3% in FL [124] 0% in FL [142] 0.8% in CLL [124] 0% in BL [124] 0% in SMZL [143] 0% in MZL [143]	*CD79A* ITAM Y188 point mutations	Not detected in multiple studies of DLBCL, FL, and other lymphoid malignancies	Inhibition of tonic BCR signaling, based on a cell line study [50]
	*CD79A* ITAM Y199 point mutations	1.8% in WM [140]	Not reported
	Complete ITAM deletions	1.5% in ABC DLBCL [48]	Increased surface BCR expression [125] Chronic active BCR signal enhancement [139]
	Other *CD79A* mutations affecting ITAM (e.g., deletions, truncations, frameshift, or splice site mutations)	6% in PCLBCL-LT (ITAM deletions affecting Y188) [131] 6.5% in ABC DLBCL [139] 1.3% in DLBCLs (deletions affecting Y188) [125] 2.9% in ABC DLBCL(splice site mutations) [48] 1.8% in WM (deletion) [140]	Increased surface BCR expression [125] Chronic active BCR signaling enhancement [139]

* For studies with available information. Abbreviations: ABC DLBCL—activated B-cell-like diffuse large B-cell lymphoma; BL—Burkitt lymphoma; CLL—chronic lymphoblastic lymphoma; DLBCL—diffuse large B-cell lymphoma; FL—follicular lymphoma; GCB DLBCL—germinal center B-cell like diffuse large B-cell lymphoma; ITAM—immunoreceptor tyrosine-based activation motif; MZL—marginal zone lymphoma; NMZL—nodal marginal zone lymphoma; PCLBCL-LT—primary cutaneous large B-cell lymphoma, leg type; SMZL—splenic marginal zone lymphoma; WM—Waldenström macroglobulinemia.

**Table 2:** Frequency of *CD79B* mutations and related B-cell receptor signaling consequences.

Frequency of Cases with Any *CD79B* ITAM Mutation	*CD79B* Mutation Subtypes	Frequency of Cases with *CD79B* Mutation Subtypes *	Signaling Consequences of *CD79B* Mutation Subtypes
65% in CLL [137,144] 56.3% in PCLBCL-LT [131] 33% in MZL [145] 0% in MZL [143] 30% in PCNSL [146] 31% in DLBCL [145] 38% in CD5^+^ DLBCLs [138] 26.8% in ABC DLBCL [57] 21.1% in ABC DLBCL [48] 10.8% in ABC DLBCL [147] 9% in ABC DLBCL [139] 23% in DLBCL [125] 14.5% in DLBCL [136] 14.3% in DLBCL [54] 14% in DLBCL [58] 12.9% in DLBCL [124] 8.5% in DLBCL [147] 8% in DLBCL [148] 5.6% in DLBCL [139] 5.1% in GCB DLBCL [147] 3.1% in GCB DLBCL [48] 1.9% in GCB DLBCL [57] 1.6% in GCB DLBCL [139] 10% in SMZL [143] 9% in WM [140] 7% in WM [149] 9% in FL [141] 5% in FL [124] 4.9% in FL [142] 0% in FL [148] 0% in FL [145] 0% in CLL [124] 0% in CLL [150] 0% in BL [48,124] 0%in MCL [148] 0% in MCL [145] 0% in marginal zone/MALT lymphoma [148] 0% gastric MALT lymphoma [48] 0% in SLL [148] 0% in CLL/SLL [145] 0% in NMZL [145]	*CD79B* ITAM Y196 point mutations	75% in transformed WM [151] 53.1% in PCLBCL-LT [131] 30% in PCNSL [146] 35% in CD5^+^ DLBCL [138] 18% in ABC DLBCL [48] 1.6% in GCB DLBCL [48] 3.7% in DLBCL [139] 10% in SMZL [143] 9% in WM [140] 8% in DLBCL [148] 3% in FL [141] 7.5% in DLBCL [147]	Surface BCR expression upregulation via inhibition of BCR internalization [48,152] Reduced BCR signaling negative regulation via decreased Lyn binding [48] Enhancement of BCR clustering [3,50] Active BCR signaling enhancement [48] NF-κB activation enhancement [48]
	*CD79B* ITAM Y207 point mutations	4% in CLL [144,153]	Surface BCR expression upregulation [152] Impairment of antigen induced BCR signaling [144,153]
	Other *CD79B* mutations affecting ITAM (e.g., deletions, truncations, frameshift, splice site/intron retention mutations)	0.7% in ABC DLBCLs (Y196 deletions) [48] 0.6% in DLBCL (deletions affecting Y207) [139] 0.5% in DLBCL (E197stop) [147] 17% in B-CLL (deletions) [144] 1.8% in DLBCL (splice site mutations and intron retention) [134]	Similar consequences as Y196 mutations
	*CD79B* ITAM mutations not affecting ITAM tyrosines	2.5% in CD5^+^ DLBCL (deletion in ITAM region before the first tyrosine) [138] 1.6% in GCB DLBCLs (L199Q) [48] 6% in FL (point mutations in ITAM) [141] 3.1% in PCLBCL-LT (E197D point mutation) [131] 31% in DLBCL [145] 1.2% in DLBCLs [139] 12.7% in DLBCL (point mutations and one frameshift) [136] 25% in CLL [137]	Not reported
	Non-ITAM mutations	Over 30% in CLL [137] 17% in CLL [144] 1.8% in DLBCL [136]	Decreased BCR expression and signaling in CLL [153]

* For studies with available information. Abbreviations: ABC DLBCL—activated B-cell like diffuse large B-cell lymphoma; BL—Burkitt lymphoma; CLL—chronic lymphoblastic lymphoma; DLBCL—diffuse large B-cell lymphoma; FL—follicular lymphoma; GCB DLBCL—germinal center B-cell like diffuse large B-cell lymphoma; ITAM—immunoreceptor tyrosine-based activation motif; MALT—mucosa-associated lymphatic tissue; MCL—mantle cell lymphoma; MZL—marginal zone lymphoma; NMZL—nodal marginal zone lymphoma; PCNSL—primary central nervous system lymphoma; PCLBCL-LT—primary cutaneous large B-cell lymphoma, leg type; SLL—small lymphocytic lymphoma; SMZL—splenic marginal zone lymphoma; WM—Waldenström macroglobulinemia.
